# The relationship between schizotypal personality features and mind wandering among college students during COVID-19 pandemic: A moderator of depression

**DOI:** 10.3389/fpsyt.2022.994082

**Published:** 2023-01-11

**Authors:** Guojun Zhao, Shuangchen Li, Qi Zhang, Xiaoxiao Guo, Fusen Xie, Shuhong Yan, Haijian Liu, Yuan Chong, Yuee Ding, Xu Li

**Affiliations:** ^1^School of Psychology, Northwest Normal University, Lanzhou, China; ^2^Academy of Plateau Science and Sustainability, Qinghai Normal University, Xining, China; ^3^School of Education, Northwest Normal University, Lanzhou, China; ^4^School of Tourism, Northwest Normal University, Lanzhou, China; ^5^Mental Health Education Center, Gansu Health Vocational College, Lanzhou, China

**Keywords:** COVID-19 pandemic, schizotypal personality features, mind wandering, depression, college student

## Abstract

**Introduction:**

Although the impact of the COVID-19 pandemic on people’s mental health has been well documented in many studies, the schizotypal personality features in the general population have not received sufficient attention.

**Methods:**

Study 1 is a longitudinal study tracking changes in schizotypal personality features among college students during the COVID-19 pandemic. A total of 153 Chinese college students were assessed using the Schizotypal Personality Questionnaire. Study 2 explored the relationship between schizotypal personality features, mind wandering, and depression. A total of 557 college students completed the Schizotypal Personality Questionnaire, the Beck Depression Inventory, and the Mind-Wandering Questionnaire during the COVID-19 pandemic.

**Results:**

Study 1 results showed that the scores from later stages in the pandemic were significantly higher than those from the initial stages on each dimension of schizotypal personality, which means that the schizotypal personality features became more obvious during the COVID-19 pandemic. Study 2 results showed that there was a positive correlation between schizotypal personality features, depression, and mind wandering.

**Discussion:**

Depression played a moderating role in the relationship between schizotypal personality features and mind wandering. The schizotypal personality features of college students increase during COVID-19; it has a positive relationship with mind wandering; depression moderates the relationship. We discussed these findings and provided some suggestions about future research.

## Introduction

On 11 March 2020, the World Health Organization (WHO) declared the outbreak of COVID-19 as a pandemic (WHO-Media-Briefing) (1). The pandemic outbreaks may pose risks to people’s mental health; both the perception of the pandemic information and social isolation are thought to lead to negative health conditions (2) and trigger a range of psychological problems (3), such as anxiety, depression, and traumatic stress disorder (4). The studies also suggested that COVID-19 could predict increased outbreaks and the prevalence of psychosis (5).

Given the effects of social isolation measures to prevent infection in people during the pandemic, we believe that schizotypal personality features in the general population are a concern. Schizotypal personality is defined as “a pervasive pattern of detachment from social relationships and a restricted range of expression of emotions in interpersonal settings” in the *Diagnostic and Statistical Manual of Mental Disorders, Fourth Edition* (DSM-IV) (6). Pedrero argued schizotypal personality features represent a “set of subclinical psychotic experiences and traits that do not reach a clinical threshold and are distributed throughout the general population” (7). Raine developed the Schizotypal Personality Questionnaire (SPQ) to assess the schizotypal personality features, and Chinese researchers demonstrated it can effectively evaluate Chinese college students’ psychological problems (8).

We speculate that these features may become salient during the COVID-19 pandemic. Some studies found that social isolation to prevent the spread of the virus caused reduced communication frequency, less social support, fewer friends, and more social anxiety (9). People were more likely to have psychiatric problems, such as somatic delusions and delusions when they were in isolation or loneliness conditions (10). When COVID-19 broke out, some scholars worried that the number of people with schizotypal personality disorders would rise (11, 12). It is worth noting that the pandemic as a stress event may also increase the likelihood of schizotypal personality features in the general population at psychiatric risk, who have certain personality features similar to those of schizotypal personality disorders (13) and are more likely to report experiencing higher levels of stress during the pandemic (14).

Not all people with schizotypal personality features experiencing stressful or major negative events eventually end up with the schizotypal personality disorder (15), however, it is more likely to lead to the onset of psychosis when having a diathesis (a biological predisposition) developing schizophrenia combined with environmental factors (e.g., the experience of a major psychosocial stressor) (16). Therefore, we chose the SPQ to examine the impact of the COVID-19 pandemic on Chinese college students. The schizotypal personality features provide a relatively comprehensive picture of how people’s mental health status changes under the influence of the pandemic (17).

Social trauma can trigger acute and transient episodes of schizotypal personality features, and college students are a population at high risk for negative mental health effects during the pandemic (18). Studies have shown that social isolation may lead to psychotic episodes (19); however, little consideration has been given to the impact of COVID-19 on susceptible populations who are at risk for psychosis (17).

Previous research has demonstrated that maladaptive personality features are significantly associated with mind wandering and that people with high schizotypal personality features in the general population experience more mind-wandering situations (20, 21). The fact that schizophrenia and mind wandering both involve reduced sensitivity to ongoing events in the external world suggests they may be closely related. Therefore, it is possible that the processing deficits associated with schizophrenia are related to levels of mind wandering (22).

Moreover, the college students’ emotion state during the pandemic should also be a concern. Some studies suggest that the relationship appears to be reciprocal: When a negative mood was induced, people’s minds also tended to wander more (23–25). Based on this evidence, Ottaviani et al. (26) suggested that the relationship between mind wandering and depression is bidirectional. Depression is the typical negative emotion that is easily triggered in the desolate condition of solitude (27).

To prevent the spread of the epidemic, Chinese colleges and universities generally adopted strict isolation measures. Students were required to stay on campus or even in dormitories, and their lives were greatly restricted. In this case, they were usually in a lonely state, which might increase the possibility of depression. Some studies have shown that the degree of social isolation during the COVID-19 pandemic is associated with depression (28). We found that a dynamic model of mind wandering can predict depression, while interventions for mind wandering have been shown to alleviate depression symptoms (29).

Emotional disorder was found to be associated with mind wandering, and inducing negative emotions could interfere with attention, increase the frequency of distraction, and reduce the possibility of participating in subsequent tasks in the condition of attention deficit (30). The likelihood of depression significantly increased during the COVID-19 pandemic (31). The previous study showed that the impairment of original switching attention indicates higher depressive symptoms, while higher depressive symptoms indicate worse selectivity and switching attention. Depressed individuals paid more attention to the stimulation with negative content than ones with neutral or positive content (32). These findings suggest a link between depression and mind wandering.

Additionally, Fontenelle et al. (33) found that negative emotions induced during the period of the COVID-19 pandemic aggravated the symptoms of schizotypal personality. A study showed that non-clinical Chinese college students with schizotypal personality features reported a higher level of depression compared with their peers (34).

Based on the above analysis, we hypothesized that the increasing schizotypal personality features would be reported by the Chinese college students in the later stages of the COVID-19 pandemic than those in the initial stages (Study 1). We also hypothesized that the schizotypal personality features would be associated with mind wandering and that the relationship would be moderated by depression.

## Study 1

### Method

#### Participants and procedure

This study was carried out in the background of the COVID-19 pandemic. We aimed to explore the impact of the pandemic on schizotypal personality features in the general population. We focus on changes in people’s mental adaptive functioning in the context of natural reactions to the COVID-19 pandemic, rather than the direct effect of infection. Hence, we recruited participants according to two criteria. The first one is that the potential participants live in areas where the severity level of the outbreak has not been identified as infectious; the second one is the potential participants themselves and their family members and friends have not contracted COVID-19. Additionally, we took into account the major physical illnesses and psychological disorders, including experience with psychotherapy, as well as conditions such as attention and depression disorders before the pandemic. We excluded these potential factors, which would affect research variables, making sure that the participants in this survey had daily living abilities and healthy living conditions before the outbreak of the COVID-19 pandemic. Due to the difficulties of sampling during the period of the epidemic, the convenience sampling method was adopted. We used an online self-reported survey, *So jump*, to collect data. We posted links to online surveys in colleges and universities in northwest China and promised to pay each participant a certain amount of money (10 Yuan). The research plan was approved by the Institutional Review Committee of Northwest Normal University. After signing the informed consent form, a total of 410 college students who engaged in online learning under the condition of home confinement volunteered to participate in the study. Any invalid questionnaires were deleted according to two criteria: (1) the questionnaires were accomplished in less than 180 s; (2) the questionnaire was not filled in at one of the two time points. The final sample comprised 153 college students. On 15 December 2019, we collected the first batch of data before the COVID-19 pandemic broke out. We asked the subjects whether they had a history of genetic diseases and conducted a simple intelligence test. On 26 November 2020, we collected the second batch of data, and the subjects were the same as those in the first batch of data. However, due to the epidemic situation, we could only take the form of an online questionnaire collection, and thus a large number of subjects were lost. Finally, our sample consists of 153 subjects.

### Measurements

#### Schizotypal Personality Questionnaire (SPQ)

We used the Schizotypal Personality Questionnaire (SPQ) to assess changes in schizotypal personality features during the COVID-19 pandemic. Its validity has been demonstrated among Chinese college students (35). The SPQ contains subscales for all nine schizotypal symptoms (17), i.e., ideas of reference, excessive social anxiety, odd beliefs or magical thinking, unusual perceptual experiences, odd or eccentric behavior, no close friends, odd speech, constricted affect, and suspiciousness. The responses of NO and YES were scored as 1 and 2, respectively. There were a total of 74 items (e.g., Do you sometimes feel that things you see on TV or read in the newspaper have a special meaning for you?). The higher the score, the more obvious the features of schizotypal personality. In this study, Cronbach’s α coefficients of the nine dimensions of schizotypal personality were in a range of 0.72–0.83, and the total Cronbach’s α coefficient was 0.87.

### Data analysis

Data were analyzed using SPSS 24.0. The paired-samples *t*-test was used to compare the differences in each dimension of SPQ between the same groups of subjects at two time points.

## Results

### Demographic data

A total of 153 participants were included in the final statistical analysis. Among them, 39.87% were male and 60.13% were female. The mean age of the samples was 21.79 years old (M_age_ = 21.79, SD = 2.76), and the age range was 18–23 years. Among them, there were 107 undergraduates (69.93%) and 46 postgraduates (30.07%).

### Paired-samples *t*-test on each dimension of schizotypal personality

The schizotypal personality features of the college students in the initial and later stages of the pandemic were assessed using the paired-samples *t*-test. The results showed that there were significant differences in the total score and dimension scores of schizotypal personality between the two time points (see Table 1), and the scores in the later stage were significantly higher than ones in the initial stage, which means that the college students’ schizotypal personality features became more prominent during the pandemic. Furthermore, we did not find any significant differences in participants’ gender and educational backgrounds on these dimensions.

**TABLE 1:** Paired-samples *t*-test on each dimension of schizotypal personality (*N* = 153).

	In the initial stage of the pandemic (M ± SD)	In the late stage of the pandemic (M ± SD)	*t*
SPQ	1.52 ± 0.16	1.75 ± 0.11	-15.91***
Ideas of reference	1.42 ± 0.22	1.62 ± 0.21	-8.20***
Unusual perceptual experiences	1.46 ± 0.25	1.66 ± 0.25	-7.06***
Odd beliefs or magical thinking	1.13 ± 0.19	1.73 ± 0.22	-24.87***
Odd or eccentric behavior	1.46 ± 0.25	1.87 ± 0.20	-15.64***
Odd speech	1.54 ± 0.21	1.76 ± 0.20	-9.58***
No close friends	1.64 ± 0.22	1.83 ± 0.18	-8.46***
Excessive social anxiety	1.44 ± 0.28	1.71 ± 0.22	-10.44***
Suspiciousness	1.57 ± 0.26	1.72 ± 0.26	-5.10***
Constricted affect	1.60 ± 0.26	1.88 ± 0.17	-11.74***

***P < 0.001.

## Discussion

We evaluated the mental adaptive functioning of the college students using the SPQ, which is a common way to understand schizotypal personality in the general population. In fact, the concept of personality and its disorders has become increasingly central to the understanding of mental illness. Hence, schizotypal personality features are used to evaluate whether college students maintain healthy psychological functions to successfully adjust to the sudden outbreak of the pandemic. The results showed that the college students’ psychological adaptive functioning became worse amidst COVID-19, with more obvious features of schizotypal personality, and the range of such a negative effect is very wide, involving the various dimensions of schizotypal personality and its total level. This is consistent with previous research on anxiety, depression, and traumatic stress in the British general population during the COVID-19 pandemic. Compared with the population before the pandemic, people during the pandemic had higher levels of anxiety, depression, and traumatic symptoms, and the prevalence of mental health problems also increased (36, 37). This has also been confirmed in a comparative study of the impact of the COVID-19 pandemic on the mental health of the German and British general populations. In this study, the researcher also used the SPQ and found that 25% of German and British respondents reported a subjective deterioration of overall mental symptoms, and 20–50% of German and British respondents reached the clinical critical value of depression and dysthymia symptoms and anxiety, indicating that a customized intervention system was needed to support most of the public (38). However, another Chinese longitudinal study showed that although the young’s psychological distress had been prevalent over the course of COVID-19, it had reduced after the peak of the pandemic (39). On the contrary, our study found the psychological disorder became more severe in the later period. One possible explanation is that the two studies used different indicators to assess psychological outcomes. Schizotypal personality features in our study are a latent and deep psychological construct and are considered a prototype characterized by impairments in identity, self-direction, empathy, and/or intimacy, along with specific maladaptive traits in the domains of psychoticism and detachment (40), which reflect the comprehensive and lasting effect of the pandemic.

Our research provides support for such a dominant conclusion and focuses more on how to prevent the adverse impact of the COVID-19 pandemic. Compared with the adult population, college students generally do not need to worry about work and economic problems when they are in social isolation. In Chinese families, parents provide for their living expenses during college, so college students rarely feel the same financial and job pressures as adults. For young students, the greatest impact of the pandemic on them may be the dramatic reduction in social communication, since social activities are the main aspect of young adult life. It is difficult for them to adapt to such changes in lifestyle.

Studying is the main task of college students, and attention is the key psychological factor to ensure the smooth progress of studying. Given that the COVID-19 pandemic has a negative influence on the psychological functioning of college students, we are interested in exploring in Study 2 whether the pandemic further predicts their mind wandering. Moreover, Study 2 examined the moderating effect of emotions, such as depression, on the relationship between schizotypal personality features and mind wandering.

## Study 2

### Method

#### Participants and procedure

This cross-sectional study was conducted on Chinese university students in the context of the COVID-19 pandemic. Data collection was conducted from 26 November 2020. Use an online, self-reported survey (*So jump*). A total of 600 college students completed the three questionnaires after signing informed consent forms, and they volunteered to participate in the study and completed the questionnaire. All questions related to the survey were managed using the *So jump* platform, and a shareable link was generated to help spread the survey across different online platforms. The online survey details informed consent, purpose, and inclusion and exclusion criteria for the study on the first page. After deleting the invalid questionnaires, the final sample consisted of 557 college students.

### Measurements

In Study 2, we adopted the same SPQ used in Study 1. Moreover, we adopted two other scales to measure mind wandering and depression.

### Mind-Wandering Questionnaire (MWQ)

The participants’ mind wandering was assessed using the MWQ, which is a self-reported questionnaire with 12 items (e.g., I have difficulty maintaining focus on simple or repetitive work) designed to assess levels of mind wandering. Items were rated on a 5-point scale ranging from 1 (almost never) to 5 (almost always). The total score was the sum of the 12 items, with higher scores indicating more mind wandering. The study from Chinese scholars demonstrated that MWQ is appropriate in the context of China (41). In this study, Cronbach’s α coefficient was 0.77.

### Beck Depression Inventory (BDI)

Depression of the participants was evaluated using the Chinese version of the BDI (42), which is a 21-item self-rated scale that evaluates key symptoms of depression. The statements provided express feelings common to depression (e.g., guilt, low self-worth, and suicidal ideation). Items are scored on a 4-point scale (1 = most and 4 = least), with total scores ranging from 21 to 84, and higher scores indicate greater depressive severity. In this study, Cronbach’s α coefficient was 0.86.

### Data analysis

Data were analyzed using SPSS 24.0. After counting some of the items in reverse, we used descriptive statistics to calculate the means and standard deviations of schizotypal personality features, mind wanderings, and depression. We also used Harman’s single-factor test to examine common method biases between the variables. We then explored the relationships between schizotypal personality features, mind wandering, and depression in the correlation analysis model. Furthermore, we used PROCESS 3.3 Model 1 to conduct multiple linear regression analysis to examine the predictive effects of schizotypal personality features and depression on mind wandering, especially the moderating effect of depression on the association by simple slope analysis.

## Results

### Demographic data

A total of 557 participants were included in the final statistical analysis. Among them, 31.80% were male and 68.20% were female, with 462 of them being undergraduate students (82.90%) and 95 of them being postgraduate students (17.10%). The ages of the participants ranged from 18 to 33 years (M_age_ = 22.00, SD = 2.86).

### Common method biases

The Harman single-factor assessment was used to test common method biases (43). All 107 items were included in an exploratory factor analysis, and the results showed that 31 factors with eigenvalues greater than 1 were obtained under the condition of non-rotation of factors, and the cumulative interpretation rate was 63.26%. Among them, the variance of the first-factor explanation was 16.14% or less than the critical value of 40%, indicating that there were no serious common method biases.

### Correlation analysis of schizotypal personality features, mind wanderings, and depression

The descriptive statistics results and correlation coefficients between the variables are presented in Table 2. The results showed that mind wandering was significantly positively correlated with schizotypal personality features as well as depression, indicating that as schizotypal personality and depression become more severe, mind wanderings are more. Meanwhile, schizotypal personality features and depression also have a significant positive correlation, which indicates that an increase in schizotypal personality is accompanied by an increase in depressive symptoms. Moreover, there was no significant gender difference on each variable except for depression (*M*_male_ ± *SD*_male_ = 0.50 ± 0.35, *M*_male_ ± *SD*_female_ = 0.44 ± 0.34, *t* = 2.16, *p* = 0.031), and there was no significant correlation between age and these variables, except for a very weak correlation with suspiciousness (*r* = 0.09, *p* = 0.044).

**TABLE 2:** Descriptive statistics and correlation analysis of schizotypal personality features, mind wanderings, and depression (*N* = 557).

	*M*	*SD*	1	2	3	4	5	6	7	8	9	19	11	12
1. MW	3.00	0.58	–											
2. D	0.46	0.34	0.42***	–										
3. SPQ	1.33	0.17	0.45***	0.56***	–									
4. IR	1.47	0.25	0.31***	0.39***	0.73***	–								
5. OBMT	1.40	0.26	0.05	0.15**	0.46***	0.41***	–							
6. UPE	1.26	0.22	0.20***	0.33***	0.66***	0.50***	0.52***	–						
7. OEB	1.20	0.26	0.29***	0.42***	0.71***	0.40***	0.24***	0.45***	–					
8. OS	1.37	0.25	0.40***	0.44***	0.76***	0.46***	0.22***	0.44***	0.58***	–				
9. NCF	1.27	0.22	0.35***	0.40***	0.67***	0.32***	0.09*	0.24***	0.45***	0.40***	–			
10. ESA	1.49	0.31	0.40***	0.38***	0.74***	0.50***	0.15**	0.33***	0.41***	0.50***	0.53***	–		
11. S	1.28	0.25	0.41***	0.50***	0.80***	0.59***	0.26***	0.41***	0.54***	0.50***	0.55***	0.54***	–	
12. CA	1.23	0.22	0.41***	0.47***	0.76***	0.40***	0.14**	0.33***	0.48***	0.62***	0.65***	0.60***	0.58***	–

*p > 0.05; **p > 0.01; ***p > 0.001.

MW, mind wandering; D, depression; SPQ, Schizotypal Personality Questionnaire; IR, ideas of reference; OBMT, odd beliefs or magical thinking; UPE, unusual perceptual experiences; OEB, odd or eccentric behavior; OS, odd speech; NCF, no close friends; ESA, excessive social anxiety; S, suspiciousness; CA, constricted affect.

### Predictive effect of SPQ on MW and moderator of depression

**FIGURE 1:**
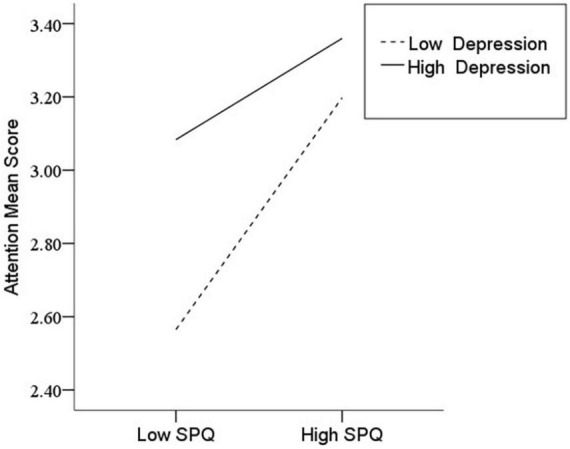
Interaction between Schizotypal Personality Questionnaire (SPQ) and depression (D) on mind wandering (MW).

**FIGURE 2:**
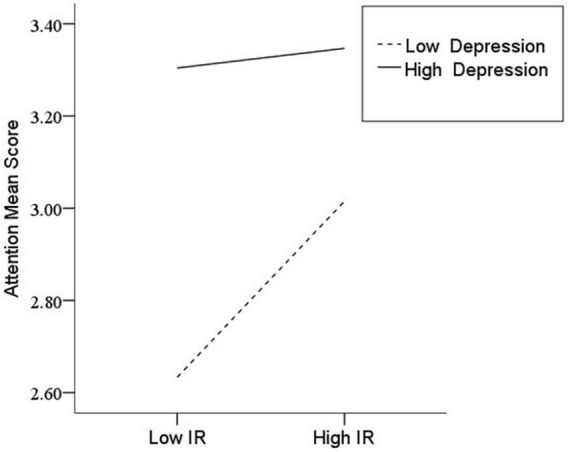
Interaction between ideas of reference (IR) and depression (D) on mind wandering (MW).

**FIGURE 3:**
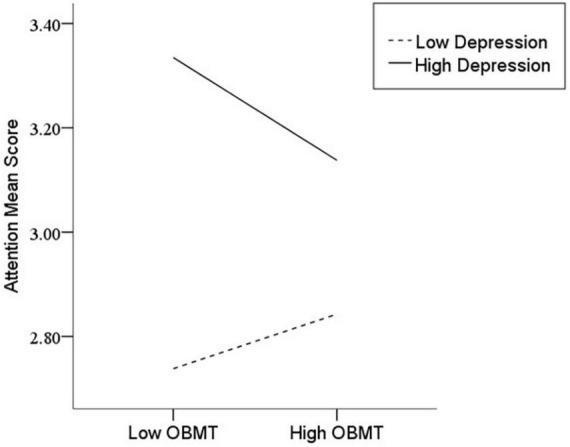
Interaction between odd beliefs or magical thinking (OBMT) and depression (D) on mind wandering (MW).

**FIGURE 4:**
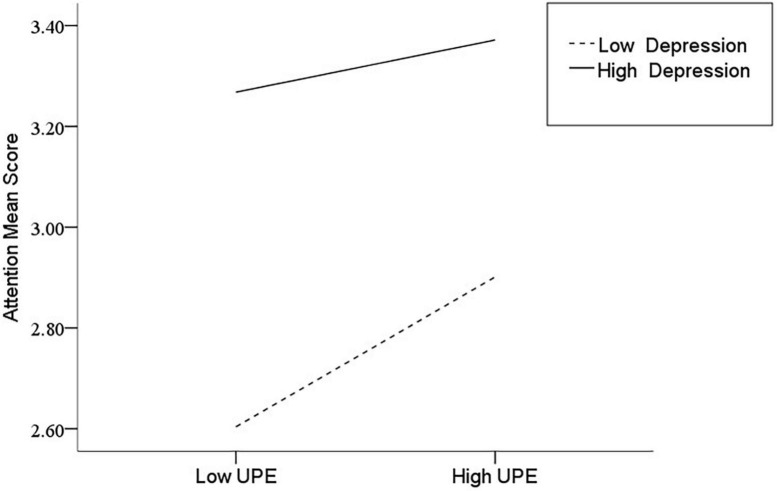
Interaction between unusual perceptual experiences (UPE) and depression (D) on mind wandering (MW).

**FIGURE 5:**
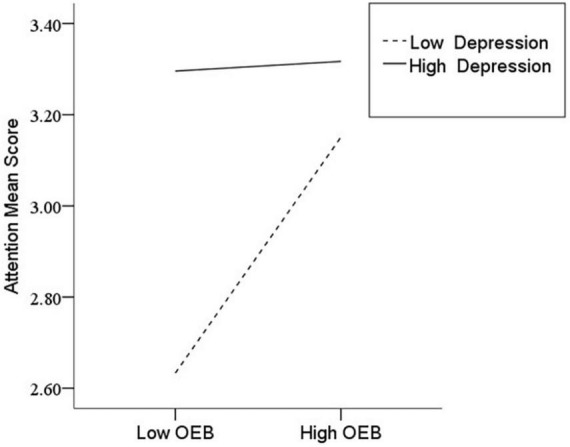
Interaction between odd or eccentric behavior (OEB) and depression (D) on mind wandering (MW).

**FIGURE 6:**
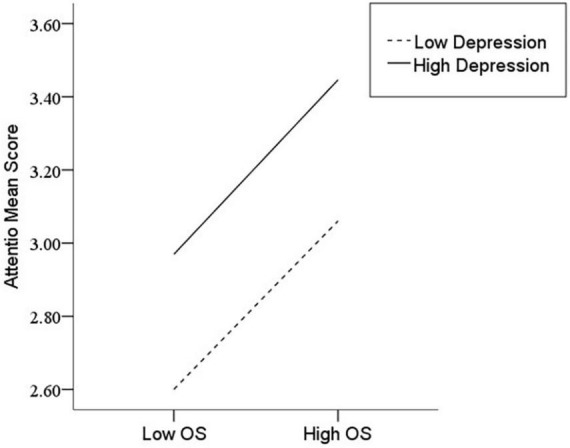
Interaction between odd speech (OS) and depression (D) on mind wandering (MW).

**FIGURE 7:**
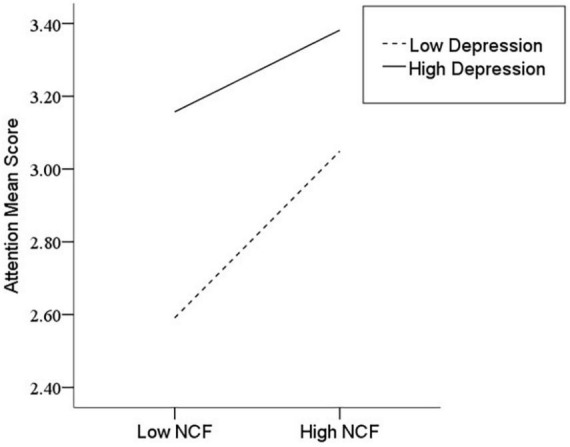
Interaction between no close friends (NCF) and depression (D) on mind wandering (MW).

**FIGURE 8:**
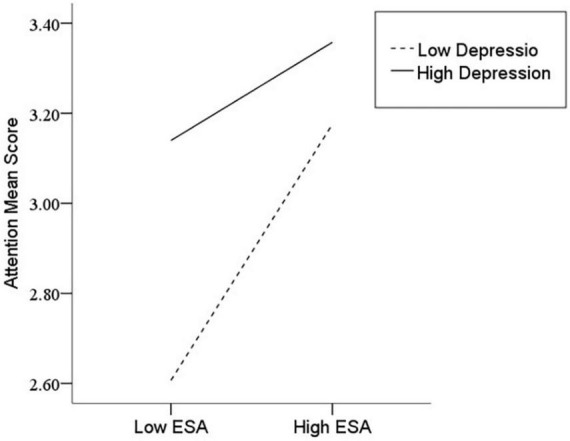
Interaction between excessive social anxiety (ESA) and depression (D) on mind wandering (MW).

**FIGURE 9:**
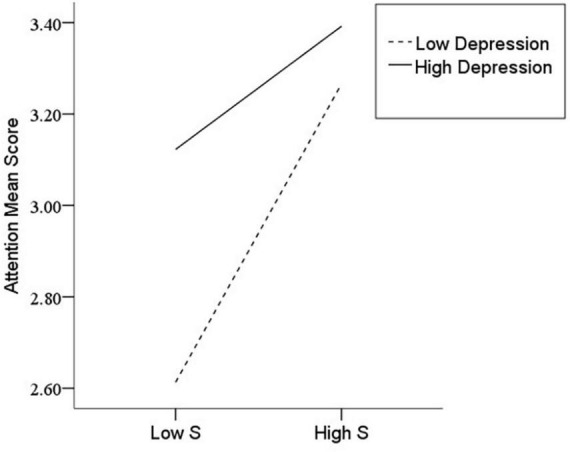
Interaction between suspiciousness (S) and depression (D) on mind wandering (MW).

**FIGURE 10:**
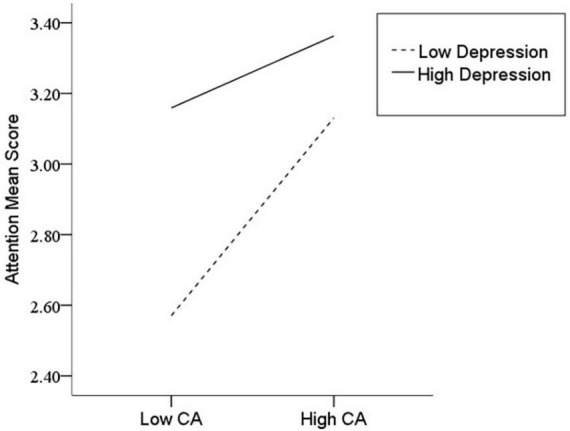
Interaction between constricted affect (CA) and depression (D) on mind wandering (MW).

After the variables were centralized, with SPQ and each of its dimensions as a separate predictive variable and MW as a dependent variable, as well as gender and age as controlled variables, we examined the 10 moderating models of depression on the relationship between SPQ and MW. All the moderating effects were demonstrated to be valid (see Table 3). Taking the SPQ as an example, the results of the PROCESS Model 1 showed that MW was significantly predicted by the SPQ and depression separately and was also predicted by their interaction. The statistic index reached the level of moderating effect, which suggests that depression has a moderating effect on the relationship between APQ and MW. Furthermore, simple slope analysis was used to test the specific mechanism of the moderator of depression. According to the standard of mean plus or minus one standard deviation, the depression score was divided into high- and low-score groups. The predictive effects of SPQ on MW for the two groups were then examined. The results showed that there were significant relationships between SPQ and MW in the low and high depression groups, but the predictive strength was different in the two groups (Figure 1). The moderating effects of depression were also demonstrated by the association between all dimensions of SPQ and MW (see Table 3 and Figures 2–10). All simple slope figures showed mind wanderings of individuals with a high depression level were more than individuals with a low depression level in both high and low groups of SPQ and subscale scores. However, the specific moderating effects showed three kinds of trends. The first kind of trend was that mind wanderings became more from the low group to the high group of SPQ and subscale scores in both high and low depression levels. However, the strength of low depression was stronger than that of high depression. Such trends include SPQ, OS, ESA, S, and CA (Figures 1, 6, 8–10). The second kind of trend showed that the higher the SPQ and subscale scores, the more mind wanderings there were for individuals with a low depression level, while there were no significant relationships for individuals with a high depression level. IR, UPE, OEB, and NCF showed such a trend (Figures 2, 4, 5, 7). The third kind of trend was the opposite of the second. The higher the SPQ and subscale scores, the fewer the mind wanderings for individuals with a high depression level, while there were no significant relationships for individuals with a low depression level. Only OBMT showed such a trend (Figure 3). It is a special case.

**TABLE 3:** Hierarchical regression analysis of Schizotypal Personality Questionnaire (SPQ) and depression (D) predicting mind wandering (MW) (*N* = 557).

	β	*t*	[BootLLCI, BootULCI]	*Δ R* ^2^	*F*	B_simple slope_		*t*
SPQ	1.00	6.88***	[0.71, 1.29]					
D	0.56	7.00***	[0.41, 0.72]					
SPQ × D	-1.53	-4.56***	[−2.16, −0.93]	0.03	20.81***	Low D	1.53	8.38***
						High D	0.48	2.52*
IR	0.37	3.86***	[0.18, 0.55]					
D	0.66	9.27***	[0.53, 0.80]					
IR × D	-0.78	-3.16**	[−1.23, −0.34]	0.01	10.00**	Low D	0.64	5.03***
						High D	0.10	0.81
OBMT	-0.03	-0.40	[−0.20.0.13]					
D	0.74	11.19***	[0.62, 0.87]					
OBMT × D	-0.61	-2.55*	[−1.06, −0.18]	0.01	6.52*	Low D	0.17	1.49
						High D	-0.24	-2.04*
UPE	0.20	1.80	[−0.03, 0.41]					
D	0.72	10.22***	[0.58, 0.86]					
UPE × D	-0.70	-2.48*	[−1.25, −0.22]	0.01	6.17*	Low D	0.44	2.84**
						High D	-0.04	-0.32
OEB	0.39	4.12***	[0.19, 0.61]					
D	0.70	9.67***	[0.56, 0.84]					
OEB × D	-0.97	-4.18***	[−1.39, −0.54]	0.02	17.47***	Low D	0.72	5.22***
						High D	0.06	0.58
OS	0.61	6.40***	[0.41, 0.79]					
D	0.59	8.12***	[0.45, 0.72]					
OS × D	-0.80	-3.17**	[−1.28, −0.32]	0.01	10.08**	Low D	0.88	6.67***
						High D	0.61	2.67**
NCF	0.62	5.74***	[0.41, 0.84]					
D	0.65	8.91***	[0.52, 0.78]					
NCF × D	-1.01	-3.40***	[−1.54, −0.50]	0.02	11.59***	Low D	0.97	6.21***
						High D	0.27	1.94
ESA	0.49	6.68***	[0.33, 0.64]					
D	0.61	8.77***	[0.48, 0.75]					
ESA × D	-0.78	-3.77***	[−1.15, −0.41]	0.02	14.22***	Low D	0.76	7.79***
						High D	0.22	2.10*
S	0.63	6.43***	[0.40, 0.84]					
D	0.58	7.73***	[0.44, 0.73]					
S × D	-0.86	-3.84***	[−1.30, −0.46]	0.02	14.76***	Low D	0.92	7.07***
						High D	0.33	2.84**
CA	0.80	7.41***	[0.58, 1.01]					
D	0.61	8.48***	[0.48, 0.74]					
CA × D	-1.42	-5.36***	[−1.90, −0.94]	0.04	28.75***	Low D	1.29	8.40***
						High D	0.80	7.41***

*P < 0.05, **P < 0.01, ***P < 0.001.

## Discussion

Our study supports the hypothesis that depression moderated the relationship between schizotypal personality features and mind wandering during the COVID-19 pandemic; specifically, the relationship between different schizotypal personality features and mind wandering differed at high and low depression levels. In previous studies, the relationships between any two variables between schizotypal personality, depression, and attention have been demonstrated. However, the relationship among the three variables has not been explored, especially for Chinese college students in the context of the COVID-19 pandemic. Thus, there is little understanding of the relationship between these variables. These limitations of previous research imply that researchers have regarded mental health as a negative outcome caused by the COVID-19 pandemic but ignored its role in predicting other psychological functions or aspects of life, such as attention, which is an essential factor in the college students’ study.

Moreover, previous research overlooked the mutual impacts of different mental health problems. In Study 2, we found that more severe schizotypal personality features and depression symptoms were accompanied by more mind wanderings; both schizotypal personality features and depression could predict mind wandering; and depression further played a moderating role in the relationship. While this result is consistent with existing research (44), it fills a gap in the literature, i.e., it demonstrates that personality features and mood have close links with cognitive ability and that different types of mental health problems have interactive effects. In this study, schizotypal personality features were regarded as the individual’s preexisting mental maladaptive functioning, while depression was regarded as a current emotional problem induced by the environment. These two kinds of mental health problems showed a complex interactional influence on cognitive qualities such as attention. With a change in the degree of depression, the association between an individual’s inherent maladaptive functioning and cognitive quality also changes. This result can more specifically explain the psychological mechanisms of individuals in the temporary social situation of coping with the COVID-19 pandemic.

### General discussion and future research directions

The COVID-19 pandemic has caused tremendous loss and trauma to people around the world, with devastation ongoing. Therefore, we need an in-depth and comprehensive understanding of the impact of this pandemic on society and people. Taking into account the life changes of the Chinese college students during the period of the COVID-19 pandemic, our research focuses on the relationship between psychological adaptive functioning (e.g., schizotypal personality features), negative mood (e.g., depression), and learning quality (e.g., mind wandering). The results show the college students’ schizotypal personality features became more obvious in the post-pandemic period than before. It indicates that students have difficulties coping with the dramatic life changes caused by the sudden outbreak of COVID-19. Schizotypal personality features, depression, and mind wandering have positive correlations with each other, indicating the worse schizotypal personality features and depression are associated with more mind wanderings. Furthermore, both schizotypal personality features and depression can predict mind wandering, and depression plays a moderating role in the relationship between schizotypal personality features and mind wandering.

According to our sampling standard, we selected the participants who live in the low-risk area and have no family members or friends who are infected with COVID-19. They appear to be in a seemingly safe state and have not been directly affected by the pandemic except for the defensive measures that limit their social interaction. However, they still face dramatic life changes, such as online learning in the condition of social isolation instead of social entertainment and interpersonal communication. Such a situation might cause difficulty in mental adaptation.

Mental health is defined as “Mental health or peace of mind is a sense of acceptance and balance that comes from having a strong system of friendship and sound social relationships that allow people to be the best they can be (45).” Sudden social conditions, such as COVID-19, will upset the balance to a certain extent. People’s social interaction is drastically reduced, and social support is reduced, which will lead to the possibility of more mental problems. Previous studies have found that individual features such as schizotypal personality features may mediate mental health outcomes related to COVID-19 (46).

Social isolation is a restrictive measure taken by many countries to control the spread of COVID-19. However, it was found that this measure has a potential negative impact on people’s mental health and adaptation functioning. Our speculation is consistent with the views of some scholars who attribute the negative effect of the pandemic on psychological outcomes to social isolation, which reduces life satisfaction and leads to mental health problems (47). Therefore, these factors may increase the risk of students developing personality disorders and emotional problems during the pandemic, as well as mind wandering. However, some researchers argued that having schizotypal personality features may be beneficial for both physical and mental health in the context of the COVID-19 pandemic because the preference for solitude by individuals with these personality features would lead to a low risk of COVID-19 transmission and could insulate them from distress given that loneliness is normally not distressing for them (46). Future studies should examine the links between schizotypal personality features and other life aspects to distinguish the different effects of schizotypal personality features.

Some studies have documented the difference between pre-COVID-19 and post-COVID-19 college students when exploring student changes during COVID-19. They considered the impact of COVID-19 on students longitudinally, and they documented changes in students’ lives as well as protective and risk factors leading to psychiatric risk. The results imply that targeted support interventions addressing life skills and improving coping in stressful situations early on may decrease the likelihood of adverse psychological outcomes in young adults later (48).

Moreover, in contrast to studies that regarded psychological problems as dependent variables, our research explored the predictive role of mental health problems on people’s life quality, such as students’ mind wandering, which may be related to their study. Moreover, we hypothesized that the COVID-19 outbreak would lead to different types of mental health problems, which have complex interactions and further affect people’s quality of life. Students are susceptible to depression, and it has been proven that stress may be a predictor thereof (49). Therefore, we suggested that the COVID-19 pandemic did not only induce students’ pre-existing personality disorder features but also cause emotional distress. In other words, personality disorder features have different relationships with students’ mind wandering under different mood conditions. Study 2 verified our hypothesis that both the preexisting mental adaptive problems of personality, such as schizotypal personality features, and temporary emotional problems, such as depression, could predict students’ mind wandering, and that emotional state can also moderate the relationship between schizotypal personality features and mind wandering.

Generally, the COVID-19 pandemic caused worse mental health conditions and further affected other aspects of life (50–52). We have only explored one unique aspect of it, and there is a long way to go.

### Limitations and future research directions

This study has several limitations. First, case study 1, although longitudinal, examined only changes in schizotypal personality, whereas case study 2 was a cross-sectional design and lacked experimental or longitudinal evidence, so no conclusions were drawn about the causal relationship between schizotypal personality and depression in terms of mind wandering; alternatively, the associations of these variables are likely to be interpreted in the opposite direction to what we would expect. Scholars have concluded that it is unclear whether mind wandering is a risk factor for depression or a consequence of depression and that there is evidence that mind wandering predicts severe depressive symptoms and severe depressive symptoms predict mind wandering (53). Therefore, future research must further define the nature of these relationships. Second, in consideration of potential factors confounding the findings, we controlled the possible influence of some key potential factors, such as physical and psychological impairment before the outbreak of the novel coronavirus pandemic and whether or not they were infected with coronavirus, and we ensured that the participants in our study could perform their daily lives and live in normal conditions. But there are still some potential factors that may confound our findings. Third, we did not further explore what effects distraction would have on students, e.g., weaker learning in online classes (54), which will be the focus of our next study. Finally, China’s anti-epidemic measures are very effective and strict, which is very different from Western countries. It is possible to find different relationships between these variables across countries. Therefore, this moderating effect needs to be supported by evidence from other countries. How to effectively maintain students’ mental and psychological health and reduce their mind wandering under a normalized epidemic will be an important new research topic.

Moreover, the underlying key factors causing mental health problems in the pandemic, such as social isolation or death anxiety, must be further explored in future research. First, as far as social isolation is concerned, in order to prevent the spread of the virus, the implementation of restrictive interpersonal communication measures would increase the chance of being alone and thus exacerbate people’s sense of loneliness. Research has found that loneliness has a negative influence on mental health and is the main factor inducing depression (55). It is necessary to consider whether social isolation is the fundamental cause of the adverse effects of the pandemic on mental health and the association between various related variables. Given that social and interpersonal relationships are the basic conditions for the existence of human society and are essential for an individual’s normal life, it is reasonable to think that the limitation of social communication would lead to mental health problems. However, there is still a lack of specific research to support this speculation. Another potential influencing factor is death anxiety. People are now more likely to feel the threat of death due to social media. Thus, could the fear of COVID-19 be the origin of mental health problems? Studies have shown that fear of death cannot only predict anxiety related to COVID-19 but also serve as a predisposing factor to explain various mental health problems (49). However, there is still a lack of empirical research on the effects of these potential factors. Therefore, subsequent researchers must further discuss these issues in the context of a pandemic, e.g., what is the fundamental cause of harm in a pandemic, the deprivation of social communication, or the fear of death? Are there other important factors that explain the negative influence of pandemics as a psychological mechanism? How do these factors influence each other? At present, the related research on the impact of the COVID-19 pandemic on people’s mental health or living conditions has not specifically distinguished the social or psychological properties of the pandemic but has broadly discussed the effect of the pandemic. Therefore, future research should pay special attention to these key variables.

## Conclusion

The COVID-19 pandemic triggers the Chinese college students’ schizotypal personality features. Both schizotypal personality features and depression can predict mind wandering, and depression has a moderating effect on the relationship between schizotypal personality features and mind wandering. These findings highlight that the negative influence of the COVID-19 pandemic on mental health is lasting and complex.

## Data availability statement

The raw data supporting the conclusions of this article will be made available by the authors, without undue reservation.

## Ethics statement

The studies involving human participants were reviewed and approved by Ethics in Human Research Committee of the School of Psychology at the Northwest Normal University in China. Written Informed consent was obtained from all individual participants included in the study.

## Author contributions

GZ and SL designed the experiments and performed the data analyses. SL, QZ, FX, HL, SY, YD, and XL recruited participants and collected the data. GZ, SL, XG, and YC wrote the manuscript. All authors contributed to the article and approved the submitted version.
